# Analysis of the intergenic sequences provided by Feria-Arroyo et al. does not support the claim of high *Borrelia burgdorferi* tick infection rates in Texas and northeastern Mexico

**DOI:** 10.1186/s13071-014-0467-9

**Published:** 2014-10-21

**Authors:** Steven J Norris, Alan G Barbour, Durland Fish, Maria A Diuk-Wasser

**Affiliations:** Departments of Pathology & Laboratory Medicine and Microbiology and Molecular Genetics, University of Texas Medical School at Houston, PO Box 20708, Houston, TX 77225-0708 USA; Departments of Microbiology & Molecular Genetics, Medicine, and Ecology & Evolutionary Biology, University of California at Irvine, Irvine, USA; Department of Epidemiology of Microbial Diseases, Yale School of Public Health, and Yale School of Forestry and Environmental Studies, New Haven, CT USA; Department of Ecology, Evolution and Environmental Sciences, Columbia University, and Department of Epidemiology of Microbial Diseases, Yale School of Public Health, New York, USA

In a recent *Parasites and Vectors* article, Feria-Arroyo et al. [1] concluded that the Texas-Mexico transboundary region is “part of a continuum in the pathogenic landscape of Lyme disease” based on their findings that 45% of *Ixodes scapularis* ticks collected from 12 locations in the region were infected with strains of *Borrelia burgdorferi* sensu stricto. In this letter, we provide evidence that the tick infection prevalence reported in this study is likely an artefact resulting from contamination of the PCR reactions with DNA from the positive control strain *B. burgdorferi* B31, as detailed below.

The assessment of tick infection rates by Feria-Arroyo et al. was based on nested PCR amplification of *flaB* and 16S rRNA-23S rRNA gene intergenic spacer (IGS) regions using DNA specimens extracted from ticks as templates and primers specific for *Borrelia* species. Of the 109 ticks characterized as *Ixodes scapularis*, 49 were said to have yielded *flaB* products consistent with *B. burgdorferi* (as indicated in Table two of the Feria-Arroyo article [1]). Twenty-two of the 49 *flaB* PCR test-positive specimens were reported to contain *B. burgdorferi* IGS sequences, which served as the basis for phylogenetic analysis (Figure three of the article [1]). *B. burgdorferi* B31, a strain first isolated from ticks collected on Shelter Island, New York in 1982 [2], was utilized as the positive control in PCR in these studies.

The results reported by Feria-Arroyo et al. [1] are contrary to all prior studies, in which the frequency of detection of *Borrelia* sequences in ticks from Texas has been less than 3%. For example, in a recent study of ticks collected from humans in Texas, Williamson et al. [3] found that only 6 ticks of 772 examined contained detectable *Borrelia* DNA. Only one of these ticks contained DNA identified as *B. burgdorferi*; this specimen was the only *I. scapularis* tick of 53 tested that gave positive results for *Borrelia* sequences [3].

Because of the incongruity of the reported results with prior studies, we contacted the authors shortly after publication and requested that the IGS and *flaB* sequences be publicly released, as is the common practice, so that the results could be examined for accuracy. At a later time, access to the chromatographs from the Sanger sequencing reactions was also requested for further verification. The IGS sequences utilized in the construction of the phylogenetic tree were made available by Feria-Arroyo et al. through the National Center of Biotechnology Information (NCBI) (http:/www.ncbi.nlm.nih.gov) on May 25, 2014. At the time of this writing, the *flaB* sequences and chromatographs have not been made available. Communications with the authors have continued in the hope of resolving the differences in interpretation of the reported results.

Independent analyses of the 21 IGS GenBank entries (KJ826414 through KJ826434) submitted to NCBI were conducted by two of us (AGB and SJN). The sequences were retrieved from GenBank database in FASTA format. For comparison, the corresponding IGS regions were also obtained from this database for the following North American *B. burgdorferi* strains: B31, 297, N40 and SGE03-1. Regions homologous to the 944 bp sequence of *B. burgdorferi* B31 between the primers Fn and Rn [4] were utilized; this region corresponds to nucleotides 443675–444618 in the B31 chromosomal sequence (AE000783.1). The sequences were combined into a single FASTA file and subjected to alignment using the Clustal Omega online program (http://www.ebi.ac.uk/Tools/msa/clustalo/). Considerable sequence heterogeneity was found at the 5’ and 3’ ends of the sequences submitted by Feria-Arroyo et al. Automated alignment of heterogeneous regions, particularly with sequence gaps, is often poor. Therefore, the alignment was refined manually. The result of this process is shown in Boxshade format in Additional file 1: Figure S1. The Clustal Omega alignments before and after manual refinement are available upon request.

All 21 of the GenBank entries had extensive sequence differences at the 5’ and 3’ ends when aligned with the B. burgdorferi B31 IGS sequence (Additional file 1: Figure S1). In the aligned region corresponding to the first 50 nt of the B31 sequence, all of the sequences reported by Feria-Arroyo et al. were truncated and many were missing additional nucleotides in comparison with the B31 sequence, resulting in gaps in the alignment. In contrast, this region is highly conserved in all available *B. burgdorferi* sequences (100% identity) and even in *B. garinii*, *B. afzelii*, *B. miyamotoi*, *B. hermsii*, and *B. parkeri* (1 nt difference in each or 98% identity; data not shown). Thus, the 5’ regions of the submitted sequences appear to contain many sequencing errors. For several of the Texas GenBank entries (KJ826414, KJ826416, KJ826417, KJ826418 and KJ826424) the sequencing errors extended further into the sequence. For example, KJ826416 has obvious sequence errors extending to nt 171 of the B31 sequence (Additional file 1: Figure S1). These error-containing regions cannot be used effectively in alignments, and were excluded from further analysis.

At the 3’ end of all the GenBank entries, there is additional sequence beyond the expected end of the generated sequence (nt 944 in the B31 sequence). These regions (at the end of Additional file 1: Figure S1) do not match any rDNA sequences. It is assumed that these sequences were ‘nonsense’ sequences generated at the 3’ end of the Fn primer-generated sequencing runs, and those also should not be used in alignments. These 3’ sequences were therefore also excluded from further analysis.

The quality of the sequences could be evaluated further by examining the Sanger sequencing chromatographs. Low signal and overlapping or atypical peaks are signs of poor sequence quality.

The parsed regions of the GenBank entries by Feria-Arroyo et al. that had no obvious sequence errors are listed in Table 1. Most of these regions corresponded to nt 49–944 in the B31 IGS sequence. All were essentially identical to the B31 sequence. For example, 10 of the GenBank entries (KJ826414, KJ826417, KJ826419, KJ826423, KJ826424, KJ826425, KJ826426, KJ826427, KJ826430 and KJ826431) had no sequence differences when the regions containing apparent sequence errors were removed. These identical regions spanned 819 to 896 nt, indicating extensive sequence identity. All but two of the remaining sequences had only 1 or 2 nt differences from the B31 sequence; KJ826416 and KJ826418, which were two of the sequences with highest 5’ end sequence errors, had only 7 and 9 nt differences, respectively (Table 1). We believe that these differences are most likely sequencing errors.

**Table 1:** **Comparison of Feria-Arroyo et al. IGS sequences with** ***B. burgdorferi*** **B31**

	**Region analyzed** ^**a**^					
**Genbank Entry No.**	**Beginning**	**End**	**Length**	**No. differences from B31**	**% Identity**	**Sequence differences (coordinate, difference)**
KJ826414	97	944	848	0	100	
KJ826415	53	813	761	1	99.9	765, A → T
KJ826416	172	835	664	7	98.9	267, G → A; 338, G → A; 691, T → A; 776, A → G; 784, T → C; 819, T → A; 829, T → A
KJ826417	127	944	818	0	100	
KJ826418	128	944	817	9	98.9	790, C → A; 836, add C; 839 A → G; 849, T → A; 902, A → C; 915 add G; 917, A → T; 921, A → T; 923, T → G
KJ826419	43	944	902	0	100	
KJ826420	49	944	896	1	99.9	930, A → −^b^
KJ826421	49	973	925	1	99.9	840, A → −
KJ826422	49	944	896	1	99.9	691, T → −
KJ826423	49	941	893	0	100	
KJ826424	74	944	871	0	100	
KJ826425	49	944	896	0	100	
KJ826426	49	941	893	0	100	
KJ826427	49	944	896	0	100	
KJ826428	49	944	896	2	99.8	903, A → T; 909 A → −
KJ826429	49	944	896	1	99.9	909, A → −
KJ826430	49	941	893	0	100	
KJ826431	49	944	896	0	100	
KJ826432	49	944	896	1	99.9	839, A → G
KJ826433	49	941	893	2	99.8	668, T → G; 887, A → G
KJ826434	49	944	896	1	99.9	920, C → G

^a^Excludes 5’ and 3’ regions with apparent sequence errors. Coordinates given are the corresponding B31 nt in the manually optimized sequence alignment.

^b^relative deletion.

Three additional reference North American *B. burgdorferi* strains were included in the alignment for comparison (Additional file 1: Figure S1). These three strains had 45, 35 and 37 nt differences from the B31 IGS sequence (Table 2), consistent with their classification as different *B. burgdorferi* genotypes [4]. These sequence differences have a completely different distribution from the sequence differences observed in the Texas GenBank entries (Table 2). Many of the sequence differences among the three reference strains are located at the same sites in the alignment, indicating consistent differences from the B31 strain. In contrast, the sequence differences in the GenBank entries by Feria-Arroyo et al. are scattered throughout the alignment, with no consistent pattern.

**Table 2:** **Sequence differences in IGS between** ***B. burgdorferi*** **B31 and other North American** ***B. burgdorferi*** **strains** ^**a**^

**B31 nt**	**B31**	**297**	**N40**	**SGE03-01**
85	T	A		
100	A	G		G
127	A	T	T	T
148	A	G	G	G
155	A	G	G	G
163	G	A	A	A
172	T	G	G	G
175	T	C	C	C
179	A	G	G	G
183	G	A	A	A
184	G	A	A	A
186	T		C	
196	T	G	G	G
295	C	T	T	T
325	T	C	C	
327	A		G	
332	G	A		
347	G		A	
360	C	G	G	G
362	A	G	G	G
366	G			A
391	A			T
409	A	G	G	G
413	G	C	C	C
422	C	T		
476	A	-		
492	T	C	C	C
496	C	T		
511	A	C		
541	G		A	A
553	G	T		
557	A	G	G	G
564	G			A
570	T			C
575	G	A	A	A
593	G		T	
602	T	G	C	C
648	T		C	
653	T	C	C	C
659	G	T	T	T
691	T	C		
730	A		C	C
739	T	G	C	C
749	C	T	T	T
759	A	G	G	G
761	C	T	T	T
783	A			G
778	C	T		
780	C	T		
806	G			T
830	G	A	A	A
868	T	-		
869	C	-		
870	T	-		
871	T	-		
872	A	-		
875	A			T
885	T	C		
No. of differences from B31		45	35	37
% Identity^b^		95.2	96.3	96.1

^a^B31 coordinates in the aligned sequences (Additional file 1: Figure S1) are indicated. A blank cell indicates that the nucleotide is the same as in B31. “**-**“ means a relative deletion.

^b^Percent identity with B31.

Based on these results, we conclude that the IGS GenBank entries associated with the Feria-Arroyo et al. article are from *B. burgdorferi* B31, most likely as the result of laboratory contamination. To give the benefit of the doubt, we can assume that the population structure of the hypothetical *B. burgdorferi* in Texas and northeastern Mexico is more similar to that of the northeastern U.S. than in the midwestern U.S. In the Northeast, the B31 genotype 1 IGS sequence was the second most prevalent genotype at 14.4% in nymphal *Ixodes scapularis*, while in the Midwest the prevalence of the IGS genotype 1 was only 6.8% [5]. Based on the 14.4% prevalence, the likelihood of finding by chance that the 20 subsequent sequences determined had the same IGS sequence as the first one found (i.e. the B31 type in this case) is 0.144^20^ = 1.5 × 10^−17^. To account for what was reported by Feria-Arroyo et al. [1], all or almost all of the *B. burgdorferi* in the ticks collected in Texas and northern Mexico would need to be clones of the same organism. Such absence of diversity has not been found in any area where *B. burgdorferi* or the other Lyme disease agents *B. afzelii* and B. *garinii* have been studied in the world, including remote Arctic and Antarctic locations. Therefore, it is not plausible that all 21 sequences arose from the tick specimens collected in Texas and exhibited such a high level of sequence identity with the B31 sequence. This finding places doubt on all of the results obtained, including the *flaB* PCR positive results reported in the article.

There are several additional concerns about the study design with regard to the collection and identification of ticks. The density of host-seeking nymphal stage *I. scapularis* infected with *B. burgdorferi* is the standard measure of Lyme disease risk [6]. All but 13 ticks from a single site reported in the study were removed from animals and were not host-seeking. Although tick stage was not reported, all 13 host-seeking ticks appear to have been collected from human subjects at deer hunter check stations. In Texas, the deer hunting season spans September 27 to February 1, which would be expected to overlap with the host-seeking activity period for adult *I. scapularis*, but not for nymphs. In addition, infection prevalence in ticks removed from animal hosts would reflect as much, if not more, the infection status of the host as it would the host-seeking tick population, since most, if not all, feeding ticks would acquire infection from a reservoir host. Therefore, the study, as designed, would not provide a clear-cut indication of the human risk for Lyme disease, even if the PCR results accurately reflected the tick infection rate.

In summary, we believe that the PCR-based results regarding the presence of *B. burgdorferi* in a high percentage of ticks collected in Texas and Mexico are not reliable for several reasons. First, the resultant IGS DNA sequences are identical or nearly identical to those of the B31 strain used as the positive control over large stretches of the PCR product length. Second, the ends of the sequences do not match any known Lyme disease *Borrelia* sequences, indicating poor quality sequence data. Third, the IGS sequences reported have little or no heterogeneity when the poor quality sequence regions are removed, which is contrary to all prior studies indicating significant heterogeneity of Lyme *Borrelia* isolates in every location sampled to date. Fourth, the authors’ phylogenetic analysis is questionable because the few IGS sequence differences that are observed in the central region of the sequences are randomly distributed and, for the reasons discussed, likely represent sequence errors. Fifth, the authors have not made the *flaB* sequence results available, and it is standard practice to make all sequences described in a publication available. In the absence of public access to the *flaB* sequences, readers are justified in concluding that the authors’ *flaB* analysis exhibits similar errors in methodology and inaccuracies in interpretation.

We support continued studies clarifying the etiology and epidemiology of Lyme disease, Southern Tick-Associated Rash Illness (STARI) and other tick-borne illnesses in Texas and the southern United States in general. However, we believe that the technical flaws in the article by Feria-Arroyo et al. obviate the potential usefulness of the reported results in illuminating the picture of tick-borne illnesses in this region. We further believe that this article provides false information that will mislead public health agencies, physicians and the public, and that it must be rectified. The information included in this letter was provided to the authors three months prior to the letter’s publication, but to date they have not made available information that addresses our concerns regarding the accuracy of the data in their article. Therefore, we have recommended retraction of the article to both the authors and the journal, in keeping with the criteria established by the Committee on Publication Ethics (http://publicationethics.org/files/retraction%20guidelines.pdf). BioMed Central, the publisher of *Parasites and Vectors*, adheres to these guidelines.

The article by Feria-Arroyo et al. has been publicized as an indication of a significant risk of Lyme disease in Texas. During the past decade, an average of 66 cases of Lyme disease in Texas has been reported to the CDC. How many cases are locally acquired has been a subject of much debate, but prior field studies on the density and infection prevalence of host-seeking nymphs of *I. scapularis* in Texas and the other Gulf Coast states indicate that the risk of *B. burgdorferi* infection is very low in these areas. The paucity of *B. burgdorferi* isolates from ticks, wildlife and humans in this region compared with the high endemicity areas in the Northeastern and North Central United States further supports this conclusion.

## Additional file

Additional file 1: Figure S1. Multiple alignment of the 16S-23S rDNA intergenic sequences reported by Feria-Arroyo et al. [1] with the corresponding intergenic sequences from *B. burgdorferi* strains B31, 297, N40, and SGE03-1. The Feria-Arroyo et al. sequences are indicated by the Genbank accession number and the designation used in the article (e.g. KJ826414 and bwtx17). The sequences were aligned using Clustal Omega, manually refined, and shaded using the program Boxshade. Nucleotides identical to the B31 sequence in the alignment are indicated by a black background and white lettering. Nonidentical sequences are indicated with a gray background (purine/purine or pyrimidine/pyrimidine) or black lettering and white background(purine/pyrimidine or pyrimidine/purine). On the consensus line, asterisks indicate identity in all the sequences.
