# Supplementary material for: Analysis of the intergenic sequences provided by Feria-Arroyo et al. does not support the claim of high Borrelia burgdorferi tick infection rates in Texas and northeastern Mexico
Source: Parasit Vectors. 2014 Oct 21;7:467. doi: 10.1186/s13071-014-0467-9 (PMC4203928; doi:10.1186/s13071-014-0467-9)
Supplement: Additional file 1: Figure S1. — Multiple alignment of the 16S-23S rDNA intergenic sequences reported by Feria-Arroyo et al. [1] with the corresponding intergenic sequences from B. burgdorferi strains B31, 297, N40, and SGE03-1. The Feria-Arroyo et al. sequences are indicated by the Genbank accession number and the designation used in the article (e.g. KJ826414 and bwtx17). The sequences were aligned using Clustal Omega, manually refined, and shaded using the program Boxshade. Nucleotides identical to the B31 sequence in the alignment are indicated by a black background and white lettering. Nonidentical sequences are indicated with a gray background (purine/purine or pyrimidine/pyrimidine) or black lettering and white background(purine/pyrimidine or pyrimidine/purine). On the consensus line, asterisks indicate identity in all the sequences. [file 13071_2014_467_MOESM1_ESM.pdf]

```
Bb_B31_IGS              1 GTAGCCGTACTGGAAAGTGCGGCTGGATCACCTCCTTTCTAAGAGAAAGATAAACTAAGG
Bb_297_IGS              1 -----CGTACTGGAAAGTGCGGCTGGATCACCTCCTTTCTAAGAGAAAGATAAACTAAGG
Bb_N40_IGS              1 GTAGCCGTACTGGAAAGTGCGGCTGGATCACCTCCTTTCTAAGAGAAAGATAAACTAAGG
Bb_SGE03-1_IGS          1 GTAGCCGTACTGGAAAGTGCGGCTGGATCACCTCCTTTCTAAGAGAAAGATAAACTAAGG
KJ826414_bwtx17         1 --------TCGGAAAAAGCCAAGCTAGTAGAGTGACAATGCGCTAGGAGCCCAGGTGTCG
KJ826415_IGRFTX4        1 ----------------------------------------------------AACTAAGG
KJ826416_bwtx32         1 --------TCGGAGA--GGCCACGCTACAGAGCTGAACTACGCTCCGAGCCCAGGTTTAT
KJ826417_bwtx23         1 ----CCGTTCGCTAGAGTAAGAGAT---------GAACTACGCTCCGAGCCCAGGTTTAT
KJ826418_bwtx31         1 ------AATCCAAGAAGTAGAGAGA---------TGACTACGCTCCGAGCTCAGGTTTAT
KJ826419_bwtx24         1 --------------------------------------CTA-GAGAAAGATAAACTAAGG
KJ826420                1 ------------AGCAGT-CGGCTGGA-CACCTCCTTTCTA-GAGAA-GATAAACTAAGG
KJ826421_GEWMA35-36     1 ------------AGCAT--CGGCTGGATCACCTCCTTTCTA-GAGAA-GATAAACTAAGG
KJ826422_GEWMA33        1 ------------AGCAGT-CGGCTGGA-CACCTCCTTTCTA-GAGAA-GATAAACTAAGG
KJ826423_CVM5           1 ---------CCGGTCACT-CGGCTGCATC-CCTCCTTTCTA-GAGAA-GATAAACTAAGG
KJ826424_KWMA11         1 ----------------GTCACTCGGATGATGGAGACCTTGATGAGTTGAGATCCGTCCGG
KJ826425_LMR2           1 ---------CGGAGCCGT-CGGCTGGATCACCTCCTTTCTA-GAGAA-GATAAACTAAGG
KJ826426_DTX            1 ------------AGCAT--CGGCTGGA-CACCTCCTTTCTA-GAGAA-GATAAACTAAGG
KJ826427_GEWMA38        1 ------------AGCAT-GCGGCTGGA-CACCTCCTTTCTA-GAGAA-GATAAACTAAGG
KJ826428_GEWMA12        1 --------CGAATACAGTGCGGCTGGATCACCTCCTTTCTA-GAGAA-GATAAACTAAGG
KJ826429_GEWMA4         1 -----------AAGCCAGTCGGCTGGA-CACCTCCTTTCTA-GAGAA-GATAAACTAAGG
KJ826430.1_GEWMA9       1 -----------AGCCAGTGCGGCTGGA-CACCTCCTTTCTA-GAGAA-GATAAACTAAGG
KJ826431_LMR1           1 ---------CGGAGCCGT-CGGCTGGATCACCTCCTTTCTA-GAGAA-GATAAACTAAGG
KJ826432_GEWMA15-16     1 -----------GAGCAGTGCGGCTGGA-CACCTCCTTTCTA-GAGAA-GATAAACTAAGG
KJ826433_GEWMA61-62     1 -------------GCAGT-CGGCTGGA-CACCTCCTTTCTA-GAGAA-GATAAACTAAGG
KJ826434_GEWMA64        1 ------------AGCCGT-CGGCTGGATCACCTCCTTTCTA-GAGAA-GATAAACTAAGG
consensus               1            ... ... ........ ............. ..... .......*....

Bb_B31_IGS             61 CTAATTCCATTAA-CTCTTCCCTACTCTTTTCTTTTGATAAGAGAGTTTTTAAAACCAGT
Bb_297_IGS             56 CTAATTCCATTAA-CTCTTCCCTACACTTTTCTTTTGATAGGAGAGTTTTTAAAACCAGT
Bb_N40_IGS             61 CTAATTCCATTAA-CTCTTCCCTACTCTTTTCTTTTGATAAGAGAGTTTTTAAAACCAGT
Bb_SGE03-1_IGS         61 CTAATTCCATTAACTCTTCCCTACTCTTTTCTTTTGATAGGAGAGTTTTTAAAACCAGT
KJ826414_bwtx17        53 CTCACACCGTTAT-GGTACACCTAACATTTTCTTTTAATAAGAGAGTTTTTAAAACCAGT
KJ826415_IGRFTX4        9 CTAATTCCATTAA-CTCTTCCCTACTCTTTTCTTTTGATAAGAGAGTTTTTAAAACCAGT
KJ826416_bwtx32        51 CACACAGGTTTAT-CGTACATCCAAGTTCGCGTGACAACTACGCGTCCGACCTCAGGTTT
KJ826417_bwtx23        48 CACA-CAGGTTCT-GGTACATCCAAGTTCGTGTGACAACTACGCGGCCGACCTCAGGTTT
KJ826418_bwtx31        46 CACA-CCGGTTTT-GGTACATCCAAGTTTGCGTGACAACTACGCGTCCGACCTCAGGTTT
KJ826419_bwtx24        22 CTAATTCCATTAA-CTCTTCCCTACTCTTTTCTTTTGATAAGAGAGTTTTTAAAACCAGT
KJ826420               45 CTAATTCCATTAA-CTCTTCCCTACTCTTTTCTTTTGATAAGAGAGTTTTTAAAACCAGT
KJ826421_GEWMA35-36    45 CTAATTCCATTAA-CTCTTCCCTACTCTTTTCTTTTGATAAGAGAGTTTTTAAAACCAGT
KJ826422_GEWMA33       45 CTAATTCCATTAA-CTCTTCCCTACTCTTTTCTTTTGATAAGAGAGTTTTTAAAACCAGT
KJ826423_CVM5          48 CTAATTCCATTAA-CTCTTCCCTACTCTTTTCTTTTGATAAGAGAGTTTTTAAAACCAGT
KJ826424_KWMA11        45 CTGATTCTCTTAC-CTCTTCCCTACTCTTTTCTTTTGATAAGAGAGTTTTTAAAACCAGT
KJ826425_LMR2          49 CTAATTCCATTAA-CTCTTCCCTACTCTTTTCTTTTGATAAGAGAGTTTTTAAAACCAGT
KJ826426_DTX           44 CTAATTCCATTAA-CTCTTCCCTACTCTTTTCTTTTGATAAGAGAGTTTTTAAAACCAGT
KJ826427_GEWMA38       45 CTAATTCCATTAA-CTCTTCCCTACTCTTTTCTTTTGATAAGAGAGTTTTTAAAACCAGT
KJ826428_GEWMA12       51 CTAATTCCATTAA-CTCTTCCCTACTCTTTTCTTTTGATAAGAGAGTTTTTAAAACCAGT
KJ826429_GEWMA4        47 CTAATTCCATTAA-CTCTTCCCTACTCTTTTCTTTTGATAAGAGAGTTTTTAAAACCAGT
KJ826430.1_GEWMA9      47 CTAATTCCATTAA-CTCTTCCCTACTCTTTTCTTTTGATAAGAGAGTTTTTAAAACCAGT
KJ826431_LMR1          49 CTAATTCCATTAA-CTCTTCCCTACTCTTTTCTTTTGATAAGAGAGTTTTTAAAACCAGT
KJ826432_GEWMA15-16    47 CTAATTCCATTAA-CTCTTCCCTACTCTTTTCTTTTGATAAGAGAGTTTTTAAAACCAGT
KJ826433_GEWMA61-62    44 CTAATTCCATTAA-CTCTTCCCTACTCTTTTCTTTTGATAAGAGAGTTTTTAAAACCAGT
KJ826434_GEWMA64       46 CTAATTCCATTAA-CTCTTCCCTACTCTTTTCTTTTGATAAGAGAGTTTTTAAAACCAGT
consensus              61 *..*.....**..........*.*...*....*....*................*....*
```

```
Bb_B31_IGS             120 GTTATTTATGTAAGTTTAAGAGAAGTTTATGTATTAAATT-------ATTGATATTAAAT
Bb_297_IGS             115 GTTATTTTTGTAAGTTTAAGAGAAGTTTGTGTATTGAATT-------TTTAATATTAAAG
Bb_N40_IGS             120 GTTATTTTTGTAAGTTTAAGAGAAGTTTGTGTATTGAATTTTTTATTTTTAATATTAAAG
Bb_SGE03-1_IGS         121 GTTATTTTTGTAAGTTTAAGAGAAGTTTGTGTATTGAATTTTTTATTTTTAATATTAAAG
KJ826414_bwtx17        112 GTTATTTATGTAAGTTTAAGAGAAGTTTATGTATTAAATT-------ATTGATATTAAAT
KJ826415_IGRFTX4        68 GTTATTTATGTAAGTTTAAGAGAAGTTTATGTATTAAATT-------ATTGATATTAAAT
KJ826416_bwtx32        110 ATCAGACTTCTTTGTTTTACATCTTTTCTGTTTTGGGTT--------GTTGATATTATTT
KJ826417_bwtx23        106 GTCAGACATGTAAGTTTAAGAGAAGTTTATGTATTAAATT-------ATTGATATTAAAT
KJ826418_bwtx31        104 ATCAGACCTGTAAGTTTAAGAGAAGTTTATGTATTAAATT-------ATTGATATTAAAT
KJ826419_bwtx24         81 GTTATTTATGTAAGTTTAAGAGAAGTTTATGTATTAAATT-------ATTGATATTAAAT
KJ826420               104 GTTATTTATGTAAGTTTAAGAGAAGTTTATGTATTAAATT-------ATTGATATTAAAT
KJ826421_GEWMA35-36    104 GTTATTTATGTAAGTTTAAGAGAAGTTTATGTATTAAATT-------ATTGATATTAAAT
KJ826422_GEWMA33       104 GTTATTTATGTAAGTTTAAGAGAAGTTTATGTATTAAATT-------ATTGATATTAAAT
KJ826423_CVM5          107 GTTATTTATGTAAGTTTAAGAGAAGTTTATGTATTAAATT-------ATTGATATTAAAT
KJ826424_KWMA11        104 GTTATTTATGTAAGTTTAAGAGAAGTTTATGTATTAAATT-------ATTGATATTAAAT
KJ826425_LMR2          108 GTTATTTATGTAAGTTTAAGAGAAGTTTATGTATTAAATT-------ATTGATATTAAAT
KJ826426_DTX           103 GTTATTTATGTAAGTTTAAGAGAAGTTTATGTATTAAATT-------ATTGATATTAAAT
KJ826427_GEWMA38       104 GTTATTTATGTAAGTTTAAGAGAAGTTTATGTATTAAATT-------ATTGATATTAAAT
KJ826428_GEWMA12       110 GTTATTTATGTAAGTTTAAGAGAAGTTTATGTATTAAATT-------ATTGATATTAAAT
KJ826429_GEWMA4        106 GTTATTTATGTAAGTTTAAGAGAAGTTTATGTATTAAATT-------ATTGATATTAAAT
KJ826430.1_GEWMA9      106 GTTATTTATGTAAGTTTAAGAGAAGTTTATGTATTAAATT-------ATTGATATTAAAT
KJ826431_LMR1          108 GTTATTTATGTAAGTTTAAGAGAAGTTTATGTATTAAATT-------ATTGATATTAAAT
KJ826432_GEWMA15-16    106 GTTATTTATGTAAGTTTAAGAGAAGTTTATGTATTAAATT-------ATTGATATTAAAT
KJ826433_GEWMA61-62    103 GTTATTTATGTAAGTTTAAGAGAAGTTTATGTATTAAATT-------ATTGATATTAAAT
KJ826434_GEWMA64       105 GTTATTTATGTAAGTTTAAGAGAAGTTTATGTATTAAATT-------ATTGATATTAAAT
consensus              121 .*.*....*.*..****.*.*....**....*.*....*.........**.******...

Bb_B31_IGS             173 AGTTTTATTAGGCTTAGACTTAATTTTAGGTCATTTTGGGGGTTTAGCTCAGTTGGCTAG
Bb_297_IGS             168 AGCTTTGTTAAACTTAGACTTAAGTTTAGGTCATTTTGGGGGTTTAGCTCAGTTGGCTAG
Bb_N40_IGS             180 AGCTTTGTTAAACCTAGACTTAAGTTTAGGTCATTTTGGGGGTTTAGCTCAGTTGGCTAG
Bb_SGE03-1_IGS         181 AGCTTTGTTAAACTTAGACTTAAGTTTAGGTCATTTTGGGGGTTTAGCTCAGTTGGCTAG
KJ826414_bwtx17        165 AGTTTTATTAGGCTTAGACTTAATTTTAGGTCATTTTGGGGGTTTAGCTCAGTTGGCTAG
KJ826415_IGRFTX4       121 AGTTTTATTAGGCTTAGACTTAATTTTAGGTCATTTTGGGGGTTTAGCTCAGTTGGCTAG
KJ826416_bwtx32        162 AGTTTTATTAGGCTTAGACTTAATTTTAGGTCATTTTGGGGGTTTAGCTCAGTTGGCTAG
KJ826417_bwtx23        159 AGTTTTATTAGGCTTAGACTTAATTTTAGGTCATTTTGGGGGTTTAGCTCAGTTGGCTAG
KJ826418_bwtx31        157 AGTTTTATTAGGCTTAGACTTAATTTTAGGTCATTTTGGGGGTTTAGCTCAGTTGGCTAG
KJ826419_bwtx24        134 AGTTTTATTAGGCTTAGACTTAATTTTAGGTCATTTTGGGGGTTTAGCTCAGTTGGCTAG
KJ826420               157 AGTTTTATTAGGCTTAGACTTAATTTTAGGTCATTTTGGGGGTTTAGCTCAGTTGGCTAG
KJ826421_GEWMA35-36    157 AGTTTTATTAGGCTTAGACTTAATTTTAGGTCATTTTGGGGGTTTAGCTCAGTTGGCTAG
KJ826422_GEWMA33       157 AGTTTTATTAGGCTTAGACTTAATTTTAGGTCATTTTGGGGGTTTAGCTCAGTTGGCTAG
KJ826423_CVM5          160 AGTTTTATTAGGCTTAGACTTAATTTTAGGTCATTTTGGGGGTTTAGCTCAGTTGGCTAG
KJ826424_KWMA11        157 AGTTTTATTAGGCTTAGACTTAATTTTAGGTCATTTTGGGGGTTTAGCTCAGTTGGCTAG
KJ826425_LMR2          161 AGTTTTATTAGGCTTAGACTTAATTTTAGGTCATTTTGGGGGTTTAGCTCAGTTGGCTAG
KJ826426_DTX           156 AGTTTTATTAGGCTTAGACTTAATTTTAGGTCATTTTGGGGGTTTAGCTCAGTTGGCTAG
KJ826427_GEWMA38       157 AGTTTTATTAGGCTTAGACTTAATTTTAGGTCATTTTGGGGGTTTAGCTCAGTTGGCTAG
KJ826428_GEWMA12       163 AGTTTTATTAGGCTTAGACTTAATTTTAGGTCATTTTGGGGGTTTAGCTCAGTTGGCTAG
KJ826429_GEWMA4        159 AGTTTTATTAGGCTTAGACTTAATTTTAGGTCATTTTGGGGGTTTAGCTCAGTTGGCTAG
KJ826430.1_GEWMA9      159 AGTTTTATTAGGCTTAGACTTAATTTTAGGTCATTTTGGGGGTTTAGCTCAGTTGGCTAG
KJ826431_LMR1          161 AGTTTTATTAGGCTTAGACTTAATTTTAGGTCATTTTGGGGGTTTAGCTCAGTTGGCTAG
KJ826432_GEWMA15-16    159 AGTTTTATTAGGCTTAGACTTAATTTTAGGTCATTTTGGGGGTTTAGCTCAGTTGGCTAG
KJ826433_GEWMA61-62    156 AGTTTTATTAGGCTTAGACTTAATTTTAGGTCATTTTGGGGGTTTAGCTCAGTTGGCTAG
KJ826434_GEWMA64       158 AGTTTTATTAGGCTTAGACTTAATTTTAGGTCATTTTGGGGGTTTAGCTCAGTTGGCTAG
consensus              181 **.***.***..*.*********.************************************
```

```
Bb_B31_IGS               233 AGCATCGGCTTTGCAAGCCGAGGGTCAAGGGTTCGAGTCCCTTAACCTCCATTGGGCTTA
Bb_297_IGS               228 AGCATCGGCTTTGCAAGCCGAGGGTCAAGGGTTCGAGTCCCTTAACCTCCATTGGGCTTA
Bb_N40_IGS               240 AGCATCGGCTTTGCAAGCCGAGGGTCAAGGGTTCGAGTCCCTTAACCTCCATTGGGCTTA
Bb_SGE03-1_IGS           241 AGCATCGGCTTTGCAAGCCGAGGGTCAAGGGTTCGAGTCCCTTAACCTCCATTGGGCTTA
KJ826414_bwtx17          225 AGCATCGGCTTTGCAAGCCGAGGGTCAAGGGTTCGAGTCCCTTAACCTCCATTGGGCTTA
KJ826415_IGRFTX4         181 AGCATCGGCTTTGCAAGCCGAGGGTCAAGGGTTCGAGTCCCTTAACCTCCATTGGGCTTA
KJ826416_bwtx32          222 AGCATCGGCTTTGCAAGCCGAGGGTCAAGGGTTCAAGTCCCTTAACCTCCATTGGGCTTA
KJ826417_bwtx23          219 AGCATCGGCTTTGCAAGCCGAGGGTCAAGGGTTCGAGTCCCTTAACCTCCATTGGGCTTA
KJ826418_bwtx31          217 AGCATCGGCTTTGCAAGCCGAGGGTCAAGGGTTCGAGTCCCTTAACCTCCATTGGGCTTA
KJ826419_bwtx24          194 AGCATCGGCTTTGCAAGCCGAGGGTCAAGGGTTCGAGTCCCTTAACCTCCATTGGGCTTA
KJ826420                 217 AGCATCGGCTTTGCAAGCCGAGGGTCAAGGGTTCGAGTCCCTTAACCTCCATTGGGCTTA
KJ826421_GEWMA35-36      217 AGCATCGGCTTTGCAAGCCGAGGGTCAAGGGTTCGAGTCCCTTAACCTCCATTGGGCTTA
KJ826422_GEWMA33         217 AGCATCGGCTTTGCAAGCCGAGGGTCAAGGGTTCGAGTCCCTTAACCTCCATTGGGCTTA
KJ826423_CVM5            220 AGCATCGGCTTTGCAAGCCGAGGGTCAAGGGTTCGAGTCCCTTAACCTCCATTGGGCTTA
KJ826424_KWMA11          217 AGCATCGGCTTTGCAAGCCGAGGGTCAAGGGTTCGAGTCCCTTAACCTCCATTGGGCTTA
KJ826425_LMR2            221 AGCATCGGCTTTGCAAGCCGAGGGTCAAGGGTTCGAGTCCCTTAACCTCCATTGGGCTTA
KJ826426_DTX             216 AGCATCGGCTTTGCAAGCCGAGGGTCAAGGGTTCGAGTCCCTTAACCTCCATTGGGCTTA
KJ826427_GEWMA38         217 AGCATCGGCTTTGCAAGCCGAGGGTCAAGGGTTCGAGTCCCTTAACCTCCATTGGGCTTA
KJ826428_GEWMA12         223 AGCATCGGCTTTGCAAGCCGAGGGTCAAGGGTTCGAGTCCCTTAACCTCCATTGGGCTTA
KJ826429_GEWMA4          219 AGCATCGGCTTTGCAAGCCGAGGGTCAAGGGTTCGAGTCCCTTAACCTCCATTGGGCTTA
KJ826430.1_GEWMA9        219 AGCATCGGCTTTGCAAGCCGAGGGTCAAGGGTTCGAGTCCCTTAACCTCCATTGGGCTTA
KJ826431_LMR1            221 AGCATCGGCTTTGCAAGCCGAGGGTCAAGGGTTCGAGTCCCTTAACCTCCATTGGGCTTA
KJ826432_GEWMA15-16      219 AGCATCGGCTTTGCAAGCCGAGGGTCAAGGGTTCGAGTCCCTTAACCTCCATTGGGCTTA
KJ826433_GEWMA61-62      216 AGCATCGGCTTTGCAAGCCGAGGGTCAAGGGTTCGAGTCCCTTAACCTCCATTGGGCTTA
KJ826434_GEWMA64         218 AGCATCGGCTTTGCAAGCCGAGGGTCAAGGGTTCGAGTCCCTTAACCTCCATTGGGCTTA
consensus                241 **********************************.*************************
```

```
Bb_B31_IGS               293 TGCCCTAAATTGTAATTAAGTATGTTTTTAAGTAACTTTGTTAAAGTAATTGTTGGAATG
Bb_297_IGS               288 TGTCCTAAATTGTAATTAAGTATGTTTTTAAGCAACTTTATTAAAGTAATTGTTGGAATG
Bb_N40_IGS               300 TGTCCTAAATTGTAATTAAGTATGTTTTTAAGCAGCTTTGTTAAAGTAATTGTTAGAATG
Bb_SGE03-1_IGS           301 TGTCCTAAATTGTAATTAAGTATGTTTTTAAGTAACTTTGTTAAAGTAATTGTTGGAATG
KJ826414_bwtx17          285 TGCCCTAAATTGTAATTAAGTATGTTTTTAAGTAACTTTGTTAAAGTAATTGTTGGAATG
KJ826415_IGRFTX4         241 TGCCCTAAATTGTAATTAAGTATGTTTTTAAGTAACTTTGTTAAAGTAATTGTTGGAATG
KJ826416_bwtx32          282 TGCCCTAAATTGTAATTAAGTATGTTTTTAAGTAACTTTGTTAAAATAATTGTTGGAATG
KJ826417_bwtx23          279 TGCCCTAAATTGTAATTAAGTATGTTTTTAAGTAACTTTGTTAAAGTAATTGTTGGAATG
KJ826418_bwtx31          277 TGCCCTAAATTGTAATTAAGTATGTTTTTAAGTAACTTTGTTAAAGTAATTGTTGGAATG
KJ826419_bwtx24          254 TGCCCTAAATTGTAATTAAGTATGTTTTTAAGTAACTTTGTTAAAGTAATTGTTGGAATG
KJ826420                 277 TGCCCTAAATTGTAATTAAGTATGTTTTTAAGTAACTTTGTTAAAGTAATTGTTGGAATG
KJ826421_GEWMA35-36      277 TGCCCTAAATTGTAATTAAGTATGTTTTTAAGTAACTTTGTTAAAGTAATTGTTGGAATG
KJ826422_GEWMA33         277 TGCCCTAAATTGTAATTAAGTATGTTTTTAAGTAACTTTGTTAAAGTAATTGTTGGAATG
KJ826423_CVM5            280 TGCCCTAAATTGTAATTAAGTATGTTTTTAAGTAACTTTGTTAAAGTAATTGTTGGAATG
KJ826424_KWMA11          277 TGCCCTAAATTGTAATTAAGTATGTTTTTAAGTAACTTTGTTAAAGTAATTGTTGGAATG
KJ826425_LMR2            281 TGCCCTAAATTGTAATTAAGTATGTTTTTAAGTAACTTTGTTAAAGTAATTGTTGGAATG
KJ826426_DTX             276 TGCCCTAAATTGTAATTAAGTATGTTTTTAAGTAACTTTGTTAAAGTAATTGTTGGAATG
KJ826427_GEWMA38         277 TGCCCTAAATTGTAATTAAGTATGTTTTTAAGTAACTTTGTTAAAGTAATTGTTGGAATG
KJ826428_GEWMA12         283 TGCCCTAAATTGTAATTAAGTATGTTTTTAAGTAACTTTGTTAAAGTAATTGTTGGAATG
KJ826429_GEWMA4          279 TGCCCTAAATTGTAATTAAGTATGTTTTTAAGTAACTTTGTTAAAGTAATTGTTGGAATG
KJ826430.1_GEWMA9        279 TGCCCTAAATTGTAATTAAGTATGTTTTTAAGTAACTTTGTTAAAGTAATTGTTGGAATG
KJ826431_LMR1            281 TGCCCTAAATTGTAATTAAGTATGTTTTTAAGTAACTTTGTTAAAGTAATTGTTGGAATG
KJ826432_GEWMA15-16      279 TGCCCTAAATTGTAATTAAGTATGTTTTTAAGTAACTTTGTTAAAGTAATTGTTGGAATG
KJ826433_GEWMA61-62      276 TGCCCTAAATTGTAATTAAGTATGTTTTTAAGTAACTTTGTTAAAGTAATTGTTGGAATG
KJ826434_GEWMA64         278 TGCCCTAAATTGTAATTAAGTATGTTTTTAAGTAACTTTGTTAAAGTAATTGTTGGAATG
consensus                301 **.*****************************.*.****.*****.********.*****
```

```
Bb_B31_IGS               353 TGAAACACAAGAAGTTAAAATTTCTGGGTTAAGTTGAGATCTGTTGATATTAAGAAAAAT
Bb_297_IGS               348 TGAAACAGAGGAAGTTAAAATTTCTGGGTTAAGTTGAGATCTGTTGATATTAAGAAGAAT
Bb_N40_IGS               360 TGAAACAGAGGAAGTTAAAATTTCTGGGTTAAGTTGAGATCTGTTGATATTAAGAAGAAT
Bb_SGE03-1_IGS           361 TGAAACAGAGGAAATTAAAATTTCTGGGTTAAGTTGAGTTCTGTTGATATTAAGAAGAAT
KJ826414_bwtx17          345 TGAAACACAAGAAGTTAAAATTTCTGGGTTAAGTTGAGATCTGTTGATATTAAGAAAAAT
KJ826415_IGRFTX4         301 TGAAACACAAGAAGTTAAAATTTCTGGGTTAAGTTGAGATCTGTTGATATTAAGAAAAAT
KJ826416_bwtx32          342 TGAAACACAAGAAGTTAAAATTTCTGGGTTAAGTTGAGATCTGTTGATATTAAGAAAAAT
KJ826417_bwtx23          339 TGAAACACAAGAAGTTAAAATTTCTGGGTTAAGTTGAGATCTGTTGATATTAAGAAAAAT
KJ826418_bwtx31          337 TGAAACACAAGAAGTTAAAATTTCTGGGTTAAGTTGAGATCTGTTGATATTAAGAAAAAT
KJ826419_bwtx24          314 TGAAACACAAGAAGTTAAAATTTCTGGGTTAAGTTGAGATCTGTTGATATTAAGAAAAAT
KJ826420                 337 TGAAACACAAGAAGTTAAAATTTCTGGGTTAAGTTGAGATCTGTTGATATTAAGAAAAAT
KJ826421_GEWMA35-36      337 TGAAACACAAGAAGTTAAAATTTCTGGGTTAAGTTGAGATCTGTTGATATTAAGAAAAAT
KJ826422_GEWMA33         337 TGAAACACAAGAAGTTAAAATTTCTGGGTTAAGTTGAGATCTGTTGATATTAAGAAAAAT
KJ826423_CVM5            340 TGAAACACAAGAAGTTAAAATTTCTGGGTTAAGTTGAGATCTGTTGATATTAAGAAAAAT
KJ826424_KWMA11          337 TGAAACACAAGAAGTTAAAATTTCTGGGTTAAGTTGAGATCTGTTGATATTAAGAAAAAT
KJ826425_LMR2            341 TGAAACACAAGAAGTTAAAATTTCTGGGTTAAGTTGAGATCTGTTGATATTAAGAAAAAT
KJ826426_DTX             336 TGAAACACAAGAAGTTAAAATTTCTGGGTTAAGTTGAGATCTGTTGATATTAAGAAAAAT
KJ826427_GEWMA38         337 TGAAACACAAGAAGTTAAAATTTCTGGGTTAAGTTGAGATCTGTTGATATTAAGAAAAAT
KJ826428_GEWMA12         343 TGAAACACAAGAAGTTAAAATTTCTGGGTTAAGTTGAGATCTGTTGATATTAAGAAAAAT
KJ826429_GEWMA4          339 TGAAACACAAGAAGTTAAAATTTCTGGGTTAAGTTGAGATCTGTTGATATTAAGAAAAAT
KJ826430.1_GEWMA9        339 TGAAACACAAGAAGTTAAAATTTCTGGGTTAAGTTGAGATCTGTTGATATTAAGAAAAAT
KJ826431_LMR1            341 TGAAACACAAGAAGTTAAAATTTCTGGGTTAAGTTGAGATCTGTTGATATTAAGAAAAAT
KJ826432_GEWMA15-16      339 TGAAACACAAGAAGTTAAAATTTCTGGGTTAAGTTGAGATCTGTTGATATTAAGAAAAAT
KJ826433_GEWMA61-62      336 TGAAACACAAGAAGTTAAAATTTCTGGGTTAAGTTGAGATCTGTTGATATTAAGAAAAAT
KJ826434_GEWMA64         338 TGAAACACAAGAAGTTAAAATTTCTGGGTTAAGTTGAGATCTGTTGATATTAAGAAAAAT
consensus                361 *******.*.***.************************.*****************.***

Bb_B31_IGS               413 GTCTAGAAGCAAAAGCAAGCTTTCGATAAAACCCGAAGTTGTTTCGCTAAAGTGCAAGGA
Bb_297_IGS               408 CTCTAGAAGTAAAAGCAAGCTTTCGATAAAACCCGAAGTTGTTTCGCTAAAGTGCAAGGA
Bb_N40_IGS               420 CTCTAGAAGCAAAAGCAAGCTTTCGATAAAACCCGAAGTTGTTTCGCTAAAGTGCAAGGA
Bb_SGE03-1_IGS           421 CTCTAGAAGCAAAAGCAAGCTTTCGATAAAACCCGAAGTTGTTTCGCTAAAGTGCAAGGA
KJ826414_bwtx17          405 GTCTAGAAGCAAAAGCAAGCTTTCGATAAAACCCGAAGTTGTTTCGCTAAAGTGCAAGGA
KJ826415_IGRFTX4         361 GTCTAGAAGCAAAAGCAAGCTTTCGATAAAACCCGAAGTTGTTTCGCTAAAGTGCAAGGA
KJ826416_bwtx32          402 GTCTAGAAGCAAAAGCAAGCTTTCGATAAAACCCGAAGTTGTTTCGCTAAAGTGCAAGGA
KJ826417_bwtx23          399 GTCTAGAAGCAAAAGCAAGCTTTCGATAAAACCCGAAGTTGTTTCGCTAAAGTGCAAGGA
KJ826418_bwtx31          397 GTCTAGAAGCAAAAGCAAGCTTTCGATAAAACCCGAAGTTGTTTCGCTAAAGTGCAAGGA
KJ826419_bwtx24          374 GTCTAGAAGCAAAAGCAAGCTTTCGATAAAACCCGAAGTTGTTTCGCTAAAGTGCAAGGA
KJ826420                 397 GTCTAGAAGCAAAAGCAAGCTTTCGATAAAACCCGAAGTTGTTTCGCTAAAGTGCAAGGA
KJ826421_GEWMA35-36      397 GTCTAGAAGCAAAAGCAAGCTTTCGATAAAACCCGAAGTTGTTTCGCTAAAGTGCAAGGA
KJ826422_GEWMA33         397 GTCTAGAAGCAAAAGCAAGCTTTCGATAAAACCCGAAGTTGTTTCGCTAAAGTGCAAGGA
KJ826423_CVM5            400 GTCTAGAAGCAAAAGCAAGCTTTCGATAAAACCCGAAGTTGTTTCGCTAAAGTGCAAGGA
KJ826424_KWMA11          397 GTCTAGAAGCAAAAGCAAGCTTTCGATAAAACCCGAAGTTGTTTCGCTAAAGTGCAAGGA
KJ826425_LMR2            401 GTCTAGAAGCAAAAGCAAGCTTTCGATAAAACCCGAAGTTGTTTCGCTAAAGTGCAAGGA
KJ826426_DTX             396 GTCTAGAAGCAAAAGCAAGCTTTCGATAAAACCCGAAGTTGTTTCGCTAAAGTGCAAGGA
KJ826427_GEWMA38         397 GTCTAGAAGCAAAAGCAAGCTTTCGATAAAACCCGAAGTTGTTTCGCTAAAGTGCAAGGA
KJ826428_GEWMA12         403 GTCTAGAAGCAAAAGCAAGCTTTCGATAAAACCCGAAGTTGTTTCGCTAAAGTGCAAGGA
KJ826429_GEWMA4          399 GTCTAGAAGCAAAAGCAAGCTTTCGATAAAACCCGAAGTTGTTTCGCTAAAGTGCAAGGA
KJ826430.1_GEWMA9        399 GTCTAGAAGCAAAAGCAAGCTTTCGATAAAACCCGAAGTTGTTTCGCTAAAGTGCAAGGA
KJ826431_LMR1            401 GTCTAGAAGCAAAAGCAAGCTTTCGATAAAACCCGAAGTTGTTTCGCTAAAGTGCAAGGA
KJ826432_GEWMA15-16      399 GTCTAGAAGCAAAAGCAAGCTTTCGATAAAACCCGAAGTTGTTTCGCTAAAGTGCAAGGA
KJ826433_GEWMA61-62      396 GTCTAGAAGCAAAAGCAAGCTTTCGATAAAACCCGAAGTTGTTTCGCTAAAGTGCAAGGA
KJ826434_GEWMA64         398 GTCTAGAAGCAAAAGCAAGCTTTCGATAAAACCCGAAGTTGTTTCGCTAAAGTGCAAGGA
consensus                421 .********.**************************************************
```

```
Bb_B31_IGS              473 TTAAACAGGATTGTATTTTTCAGCAGCCTATTTTATAAACGATCTGCATTTAGTAAATAG
Bb_297_IGS              468 TTA-ACAGGATTGTATTTTCCAGTAGCCTATTTTATAACCGATCTGCATTTAGTAAATAG
Bb_N40_IGS              480 TTAAACAGGATTGTATTTTCCAGCAGCCTATTTTATAAACGATCTGCATTTAGTAAATAG
Bb_SGE03-1_IGS          481 TTAAACAGGATTGTATTTTCCAGCAGCCTATTTTATAAACGATCTGCATTTAGTAAATAG
KJ826414_bwtx17         465 TTAAACAGGATTGTATTTTTCAGCAGCCTATTTTATAAACGATCTGCATTTAGTAAATAG
KJ826415_IGRFTX4        421 TTAAACAGGATTGTATTTTTCAGCAGCCTATTTTATAAACGATCTGCATTTAGTAAATAG
KJ826416_bwtx32         462 TTAAACAGGATTGTATTTTTCAGCAGCCTATTTTATAAACGATCTGCATTTAGTAAATAG
KJ826417_bwtx23         459 TTAAACAGGATTGTATTTTTCAGCAGCCTATTTTATAAACGATCTGCATTTAGTAAATAG
KJ826418_bwtx31         457 TTAAACAGGATTGTATTTTTCAGCAGCCTATTTTATAAACGATCTGCATTTAGTAAATAG
KJ826419_bwtx24         434 TTAAACAGGATTGTATTTTTCAGCAGCCTATTTTATAAACGATCTGCATTTAGTAAATAG
KJ826420                457 TTAAACAGGATTGTATTTTTCAGCAGCCTATTTTATAAACGATCTGCATTTAGTAAATAG
KJ826421_GEWMA35-36     457 TTAAACAGGATTGTATTTTTCAGCAGCCTATTTTATAAACGATCTGCATTTAGTAAATAG
KJ826422_GEWMA33        457 TTAAACAGGATTGTATTTTTCAGCAGCCTATTTTATAAACGATCTGCATTTAGTAAATAG
KJ826423_CVM5           460 TTAAACAGGATTGTATTTTTCAGCAGCCTATTTTATAAACGATCTGCATTTAGTAAATAG
KJ826424_KWMA11         457 TTAAACAGGATTGTATTTTTCAGCAGCCTATTTTATAAACGATCTGCATTTAGTAAATAG
KJ826425_LMR2           461 TTAAACAGGATTGTATTTTTCAGCAGCCTATTTTATAAACGATCTGCATTTAGTAAATAG
KJ826426_DTX            456 TTAAACAGGATTGTATTTTTCAGCAGCCTATTTTATAAACGATCTGCATTTAGTAAATAG
KJ826427_GEWMA38        457 TTAAACAGGATTGTATTTTTCAGCAGCCTATTTTATAAACGATCTGCATTTAGTAAATAG
KJ826428_GEWMA12        463 TTAAACAGGATTGTATTTTTCAGCAGCCTATTTTATAAACGATCTGCATTTAGTAAATAG
KJ826429_GEWMA4         459 TTAAACAGGATTGTATTTTTCAGCAGCCTATTTTATAAACGATCTGCATTTAGTAAATAG
KJ826430.1_GEWMA9       459 TTAAACAGGATTGTATTTTTCAGCAGCCTATTTTATAAACGATCTGCATTTAGTAAATAG
KJ826431_LMR1           461 TTAAACAGGATTGTATTTTTCAGCAGCCTATTTTATAAACGATCTGCATTTAGTAAATAG
KJ826432_GEWMA15-16     459 TTAAACAGGATTGTATTTTTCAGCAGCCTATTTTATAAACGATCTGCATTTAGTAAATAG
KJ826433_GEWMA61-62     456 TTAAACAGGATTGTATTTTTCAGCAGCCTATTTTATAAACGATCTGCATTTAGTAAATAG
KJ826434_GEWMA64        458 TTAAACAGGATTGTATTTTTCAGCAGCCTATTTTATAAACGATCTGCATTTAGTAAATAG
consensus               481 ***.***************.***.**************.*********************
```

```
Bb_B31_IGS              533 TTTTTAGTTAGGAAATAATGTAGATTACTAAGTGTGATGTCTGAGAGAAGGACAAGTATT
Bb_297_IGS              527 TTTTTAGTTAGGAAATAATGTATATTGCTAAGTGTGATGTCTGAAAGAAGGACAAGTATT
Bb_N40_IGS              540 TTTTTAGTTAAGAAATAATGTAGATTGCTAAGTGTGATGTCTGAAAGAAGGACAAGTATT
Bb_SGE03-1_IGS          541 TTTTTAGTTAAGAAATAATGTAGATTGCTAAGTATGATGCCTGAAAGAAGGACAAGTATT
KJ826414_bwtx17         525 TTTTTAGTTAGGAAATAATGTAGATTACTAAGTGTGATGTCTGAGAGAAGGACAAGTATT
KJ826415_IGRFTX4        481 TTTTTAGTTAGGAAATAATGTAGATTACTAAGTGTGATGTCTGAGAGAAGGACAAGTATT
KJ826416_bwtx32         522 TTTTTAGTTAGGAAATAATGTAGATTACTAAGTGTGATGTCTGAGAGAAGGACAAGTATT
KJ826417_bwtx23         519 TTTTTAGTTAGGAAATAATGTAGATTACTAAGTGTGATGTCTGAGAGAAGGACAAGTATT
KJ826418_bwtx31         517 TTTTTAGTTAGGAAATAATGTAGATTACTAAGTGTGATGTCTGAGAGAAGGACAAGTATT
KJ826419_bwtx24         494 TTTTTAGTTAGGAAATAATGTAGATTACTAAGTGTGATGTCTGAGAGAAGGACAAGTATT
KJ826420                517 TTTTTAGTTAGGAAATAATGTAGATTACTAAGTGTGATGTCTGAGAGAAGGACAAGTATT
KJ826421_GEWMA35-36     517 TTTTTAGTTAGGAAATAATGTAGATTACTAAGTGTGATGTCTGAGAGAAGGACAAGTATT
KJ826422_GEWMA33        517 TTTTTAGTTAGGAAATAATGTAGATTACTAAGTGTGATGTCTGAGAGAAGGACAAGTATT
KJ826423_CVM5           520 TTTTTAGTTAGGAAATAATGTAGATTACTAAGTGTGATGTCTGAGAGAAGGACAAGTATT
KJ826424_KWMA11         517 TTTTTAGTTAGGAAATAATGTAGATTACTAAGTGTGATGTCTGAGAGAAGGACAAGTATT
KJ826425_LMR2           521 TTTTTAGTTAGGAAATAATGTAGATTACTAAGTGTGATGTCTGAGAGAAGGACAAGTATT
KJ826426_DTX            516 TTTTTAGTTAGGAAATAATGTAGATTACTAAGTGTGATGTCTGAGAGAAGGACAAGTATT
KJ826427_GEWMA38        517 TTTTTAGTTAGGAAATAATGTAGATTACTAAGTGTGATGTCTGAGAGAAGGACAAGTATT
KJ826428_GEWMA12        523 TTTTTAGTTAGGAAATAATGTAGATTACTAAGTGTGATGTCTGAGAGAAGGACAAGTATT
KJ826429_GEWMA4         519 TTTTTAGTTAGGAAATAATGTAGATTACTAAGTGTGATGTCTGAGAGAAGGACAAGTATT
KJ826430.1_GEWMA9       519 TTTTTAGTTAGGAAATAATGTAGATTACTAAGTGTGATGTCTGAGAGAAGGACAAGTATT
KJ826431_LMR1           521 TTTTTAGTTAGGAAATAATGTAGATTACTAAGTGTGATGTCTGAGAGAAGGACAAGTATT
KJ826432_GEWMA15-16     519 TTTTTAGTTAGGAAATAATGTAGATTACTAAGTGTGATGTCTGAGAGAAGGACAAGTATT
KJ826433_GEWMA61-62     516 TTTTTAGTTAGGAAATAATGTAGATTACTAAGTGTGATGTCTGAGAGAAGGACAAGTATT
KJ826434_GEWMA64        518 TTTTTAGTTAGGAAATAATGTAGATTACTAAGTGTGATGTCTGAGAGAAGGACAAGTATT
consensus               541 **********.***********.***.******.*****.*****.**************
```

```
Bb_B31_IGS             593 GTAGCGAGCTTAAATCCTTATTATCGTTGCCAGTATTTAGTGG-TAGGGATTCGGATAAG
Bb_297_IGS             587 GTAGCGAGCGTAAATCCTTGTTATCGTTGCCAGTATTTAGTGG-TAGGGATTCGGATAAG
Bb_N40_IGS             600 TTAGCGAGCCTAAATCCTTGTTATCGTTGCCAGTATTTAGTGG-TAGGGATTCGGACAAG
Bb_SGE03-1_IGS         601 GTAGCGAGCCTAAATCCTTGTTATCGTTGCCAGTATTTAGTGG-TAGGGATTCGGATAAG
KJ826414_bwtx17        585 GTAGCGAGCTTAAATCCTTATTATCGTTGCCAGTATTTAGTGG-TAGGGATTCGGATAAG
KJ826415_IGRFTX4       541 GTAGCGAGCTTAAATCCTTATTATCGTTGCCAGTATTTAGTGG-TAGGGATTCGGATAAG
KJ826416_bwtx32        582 GTAGCGAGCTTAAATCCTTATTATCGTTGCCAGTATTTAGTGG-TAGGGATTCGGATAAG
KJ826417_bwtx23        579 GTAGCGAGCTTAAATCCTTATTATCGTTGCCAGTATTTAGTGG-TAGGGATTCGGATAAG
KJ826418_bwtx31        577 GTAGCGAGCTTAAATCCTTATTATCGTTGCCAGTATTTAGTGG-TAGGGATTCGGATAAG
KJ826419_bwtx24        554 GTAGCGAGCTTAAATCCTTATTATCGTTGCCAGTATTTAGTGG-TAGGGATTCGGATAAG
KJ826420               577 GTAGCGAGCTTAAATCCTTATTATCGTTGCCAGTATTTAGTGG-TAGGGATTCGGATAAG
KJ826421_GEWMA35-36    577 GTAGCGAGCTTAAATCCTTATTATCGTTGCCAGTATTTAGTGG-TAGGGATTCGGATAAG
KJ826422_GEWMA33       577 GTAGCGAGCTTAAATCCTTATTATCGTTGCCAGTATTTAGTGG-TAGGGATTCGGATAAG
KJ826423_CVM5          580 GTAGCGAGCTTAAATCCTTATTATCGTTGCCAGTATTTAGTGG-TAGGGATTCGGATAAG
KJ826424_KWMA11        577 GTAGCGAGCTTAAATCCTTATTATCGTTGCCAGTATTTAGTGG-TAGGGATTCGGATAAG
KJ826425_LMR2          581 GTAGCGAGCTTAAATCCTTATTATCGTTGCCAGTATTTAGTGG-TAGGGATTCGGATAAG
KJ826426_DTX           576 GTAGCGAGCTTAAATCCTTATTATCGTTGCCAGTATTTAGTGG-TAGGGATTCGGATAAG
KJ826427_GEWMA38       577 GTAGCGAGCTTAAATCCTTATTATCGTTGCCAGTATTTAGTGG-TAGGGATTCGGATAAG
KJ826428_GEWMA12       583 GTAGCGAGCTTAAATCCTTATTATCGTTGCCAGTATTTAGTGG-TAGGGATTCGGATAAG
KJ826429_GEWMA4        579 GTAGCGAGCTTAAATCCTTATTATCGTTGCCAGTATTTAGTGG-TAGGGATTCGGATAAG
KJ826430.1_GEWMA9      579 GTAGCGAGCTTAAATCCTTATTATCGTTGCCAGTATTTAGTGG-TAGGGATTCGGATAAG
KJ826431_LMR1          581 GTAGCGAGCTTAAATCCTTATTATCGTTGCCAGTATTTAGTGG-TAGGGATTCGGATAAG
KJ826432_GEWMA15-16    579 GTAGCGAGCTTAAATCCTTATTATCGTTGCCAGTATTTAGTGG-TAGGGATTCGGATAAG
KJ826433_GEWMA61-62    576 GTAGCGAGCTTAAATCCTTATTATCGTTGCCAGTATTTAGTGGGTAGGGATTCGGATAAG
KJ826434_GEWMA64       578 GTAGCGAGCTTAAATCCTTATTATCGTTGCCAGTATTTAGTGG-TAGGGATTCGGATAAG
consensus              601 .********.*********.***********************.************.***

Bb_B31_IGS             652 ATTGCCAGTTATAAGT-TGGAGGAAGGCAAAGATTGCATTAAATCGTTATCGCTCTTATG
Bb_297_IGS             646 ACTGCCATTTATAAGT-TGGAGGAAGGCAAAGATTGCATCAAATCGTTATCGCTCTTATG
Bb_N40_IGS             659 ACTGCCATTTATAAGT-TGGAGGAAGGCAAAGATTGCATTAAATCGTTATCGCTCTTATG
Bb_SGE03-1_IGS         660 ACTGCCATTTATAAGT-TGGAGGAAGGCAAAGATTGCATTAAATCGTTATCGCTCTTATG
KJ826414_bwtx17        644 ATTGCCAGTTATAAGT-TGGAGGAAGGCAAAGATTGCATTAAATCGTTATCGCTCTTATG
KJ826415_IGRFTX4       600 ATTGCCAGTTATAAGT-TGGAGGAAGGCAAAGATTGCATTAAATCGTTATCGCTCTTATG
KJ826416_bwtx32        641 ATTGCCAGTTATAAGT-TGGAGGAAGGCAAAGATTGCATTAAATCGATATCGCTCTTATG
KJ826417_bwtx23        638 ATTGCCAGTTATAAGT-TGGAGGAAGGCAAAGATTGCATTAAATCGTTATCGCTCTTATG
KJ826418_bwtx31        636 ATTGCCAGTTATAAGT-TGGAGGAAGGCAAAGATTGCATTAAATCGTTATCGCTCTTATG
KJ826419_bwtx24        613 ATTGCCAGTTATAAGT-TGGAGGAAGGCAAAGATTGCATTAAATCGTTATCGCTCTTATG
KJ826420               636 ATTGCCAGTTATAAGT-TGGAGGAAGGCAAAGATTGCATTAAATCGTTATCGCTCTTATG
KJ826421_GEWMA35-36    636 ATTGCCAGTTATAAGT-TGGAGGAAGGCAAAGATTGCATTAAATCGTTATCGCTCTTATG
KJ826422_GEWMA33       636 ATTGCCAGTTATAAGT-TGGAGGAAGGCAAAGATTGCATT-AATCGTTATCGCTCTTATG
KJ826423_CVM5          639 ATTGCCAGTTATAAGT-TGGAGGAAGGCAAAGATTGCATTAAATCGTTATCGCTCTTATG
KJ826424_KWMA11        636 ATTGCCAGTTATAAGT-TGGAGGAAGGCAAAGATTGCATTAAATCGTTATCGCTCTTATG
KJ826425_LMR2          640 ATTGCCAGTTATAAGT-TGGAGGAAGGCAAAGATTGCATTAAATCGTTATCGCTCTTATG
KJ826426_DTX           635 ATTGCCAGTTATAAGT-TGGAGGAAGGCAAAGATTGCATTAAATCGTTATCGCTCTTATG
KJ826427_GEWMA38       636 ATTGCCAGTTATAAGT-TGGAGGAAGGCAAAGATTGCATTAAATCGTTATCGCTCTTATG
KJ826428_GEWMA12       642 ATTGCCAGTTATAAGT-TGGAGGAAGGCAAAGATTGCATTAAATCGTTATCGCTCTTATG
KJ826429_GEWMA4        638 ATTGCCAGTTATAAGT-TGGAGGAAGGCAAAGATTGCATTAAATCGTTATCGCTCTTATG
KJ826430.1_GEWMA9      638 ATTGCCAGTTATAAGT-TGGAGGAAGGCAAAGATTGCATTAAATCGTTATCGCTCTTATG
KJ826431_LMR1          640 ATTGCCAGTTATAAGT-TGGAGGAAGGCAAAGATTGCATTAAATCGTTATCGCTCTTATG
KJ826432_GEWMA15-16    638 ATTGCCAGTTATAAGT-TGGAGGAAGGCAAAGATTGCATTAAATCGTTATCGCTCTTATG
KJ826433_GEWMA61-62    636 ATTGCCAGTTATAAGTTGGGAGGAAGGCAAAGATTGCATTAAATCGTTATCGCTCTTATG
KJ826434_GEWMA64       637 ATTGCCAGTTATAAGT-TGGAGGAAGGCAAAGATTGCATTAAATCGTTATCGCTCTTATG
consensus              661 *.*****.********..*********************..*****.*************
```

```
Bb_B31_IGS            711 TTTTAGGTTACAAGTTTGCAACAATAACTCAAAAAAGCCAAGCAAAATACCATAAAGCAG
Bb_297_IGS            705 TTTTAGGTTACAAGTTTGCAACAATAACGCAAAAAAGCTAAGCAAAATGCTATAAAGCAG
Bb_N40_IGS            718 TTTTAGGTTACAAGTTTGCCACAATAACCCAAAAAAGCTAAGCAAAATGCTATAAAGCAG
Bb_SGE03-1_IGS        719 TTTTAGGTTACAAGTTTGCCACAATAACCCAAAAAAGCTAAGCAAAATGCTATAAAGCAG
KJ826414_bwtx17       703 TTTTAGGTTACAAGTTTGCAACAATAACTCAAAAAAGCCAAGCAAAATACCATAAAGCAG
KJ826415_IGRFTX4      659 TTTTAGGTTACAAGTTTGCAACAATAACTCAAAAAAGCCAAGCAAAATACCATATAGCAG
KJ826416_bwtx32       700 TTTTAGGTTACAAGTTTGCAACAATAACTCAAAAAAGCCAAGCAAAATACCATAAAGCAG
KJ826417_bwtx23       697 TTTTAGGTTACAAGTTTGCAACAATAACTCAAAAAAGCCAAGCAAAATACCATAAAGCAG
KJ826418_bwtx31       695 TTTTAGGTTACAAGTTTGCAACAATAACTCAAAAAAGCCAAGCAAAATACCATAAAGCAG
KJ826419_bwtx24       672 TTTTAGGTTACAAGTTTGCAACAATAACTCAAAAAAGCCAAGCAAAATACCATAAAGCAG
KJ826420              695 TTTTAGGTTACAAGTTTGCAACAATAACTCAAAAAAGCCAAGCAAAATACCATAAAGCAG
KJ826421_GEWMA35-36   695 TTTTAGGTTACAAGTTTGCAACAATAACTCAAAAAAGCCAAGCAAAATACCATAAAGCAG
KJ826422_GEWMA33      694 TTTTAGGTTACAAGTTTGCAACAATAACTCAAAAAAGCCAAGCAAAATACCATAAAGCAG
KJ826423_CVM5         698 TTTTAGGTTACAAGTTTGCAACAATAACTCAAAAAAGCCAAGCAAAATACCATAAAGCAG
KJ826424_KWMA11       695 TTTTAGGTTACAAGTTTGCAACAATAACTCAAAAAAGCCAAGCAAAATACCATAAAGCAG
KJ826425_LMR2         699 TTTTAGGTTACAAGTTTGCAACAATAACTCAAAAAAGCCAAGCAAAATACCATAAAGCAG
KJ826426_DTX          694 TTTTAGGTTACAAGTTTGCAACAATAACTCAAAAAAGCCAAGCAAAATACCATAAAGCAG
KJ826427_GEWMA38      695 TTTTAGGTTACAAGTTTGCAACAATAACTCAAAAAAGCCAAGCAAAATACCATAAAGCAG
KJ826428_GEWMA12      701 TTTTAGGTTACAAGTTTGCAACAATAACTCAAAAAAGCCAAGCAAAATACCATAAAGCAG
KJ826429_GEWMA4       697 TTTTAGGTTACAAGTTTGCAACAATAACTCAAAAAAGCCAAGCAAAATACCATAAAGCAG
KJ826430.1_GEWMA9     697 TTTTAGGTTACAAGTTTGCAACAATAACTCAAAAAAGCCAAGCAAAATACCATAAAGCAG
KJ826431_LMR1         699 TTTTAGGTTACAAGTTTGCAACAATAACTCAAAAAAGCCAAGCAAAATACCATAAAGCAG
KJ826432_GEWMA15-16   697 TTTTAGGTTACAAGTTTGCAACAATAACTCAAAAAAGCCAAGCAAAATACCATAAAGCAG
KJ826433_GEWMA61-62   696 TTTTAGGTTACAAGTTTGCAACAATAACTCAAAAAAGCCAAGCAAAATACCATAAAGCAG
KJ826434_GEWMA64      696 TTTTAGGTTACAAGTTTGCAACAATAACTCAAAAAAGCCAAGCAAAATACCATAAAGCAG
consensus             721 *******************.********.*********.*********.*.***.*****

Bb_B31_IGS            771 ATTTCAGTTTGGATTTGCCCGACACTCAATGGCATGAAGTTGTAATTGTTAGTAATCGTG
Bb_297_IGS            765 ATTTCAGTTTGGATTTGTCTGACACTCAATGGCATGAAGTTGTAATTGTTAGTAATCGTA
Bb_N40_IGS            778 ATTTCAGTTTGGATTTGCCCGACACTCAATGGCATGAAGTTGTAATTGTTAGTAATCGTA
Bb_SGE03-1_IGS        779 ATTTCAGTTTGGGTTTGCCCGACACTCAATGGCATTAAGTTGTAATTGTTAGTAATCGTA
KJ826414_bwtx17       763 ATTTCAGTTTGGATTTGCCCGACACTCAATGGCATGAAGTTGTAATTGTTAGTAATCGTG
KJ826415_IGRFTX4      719 ATTTCAGTTTGGATTTGCCCGACACTCAATGGCATGAAGTTGT-----------------
KJ826416_bwtx32       760 ATTTCGGTTTGGACTTGCCCGACACTCAATGGCATGAAGTTGTAATTGATAGTAATCGAG
KJ826417_bwtx23       757 ATTTCAGTTTGGATTTGCCCGACACTCAATGGCATGAAGTTGTAATTGTTAGTAATCGTG
KJ826418_bwtx31       755 ATTTCAGTTTGGATTTGCCAGACACTCAATGGCATGAAGTTGTAATTGTTAGTAATCGTG
KJ826419_bwtx24       732 ATTTCAGTTTGGATTTGCCCGACACTCAATGGCATGAAGTTGTAATTGTTAGTAATCGTG
KJ826420              755 ATTTCAGTTTGGATTTGCCCGACACTCAATGGCATGAAGTTGTAATTGTTAGTAATCGTG
KJ826421_GEWMA35-36   755 ATTTCAGTTTGGATTTGCCCGACACTCAATGGCATGAAGTTGTAATTGTTAGTAATCGTG
KJ826422_GEWMA33      754 ATTTCAGTTTGGATTTGCCCGACACTCAATGGCATGAAGTTGTAATTGTTAGTAATCGTG
KJ826423_CVM5         758 ATTTCAGTTTGGATTTGCCCGACACTCAATGGCATGAAGTTGTAATTGTTAGTAATCGTG
KJ826424_KWMA11       755 ATTTCAGTTTGGATTTGCCCGACACTCAATGGCATGAAGTTGTAATTGTTAGTAATCGTG
KJ826425_LMR2         759 ATTTCAGTTTGGATTTGCCCGACACTCAATGGCATGAAGTTGTAATTGTTAGTAATCGTG
KJ826426_DTX          754 ATTTCAGTTTGGATTTGCCCGACACTCAATGGCATGAAGTTGTAATTGTTAGTAATCGTG
KJ826427_GEWMA38      755 ATTTCAGTTTGGATTTGCCCGACACTCAATGGCATGAAGTTGTAATTGTTAGTAATCGTG
KJ826428_GEWMA12      761 ATTTCAGTTTGGATTTGCCCGACACTCAATGGCATGAAGTTGTAATTGTTAGTAATCGTG
KJ826429_GEWMA4       757 ATTTCAGTTTGGATTTGCCCGACACTCAATGGCATGAAGTTGTAATTGTTAGTAATCGTG
KJ826430.1_GEWMA9     757 ATTTCAGTTTGGATTTGCCCGACACTCAATGGCATGAAGTTGTAATTGTTAGTAATCGTG
KJ826431_LMR1         759 ATTTCAGTTTGGATTTGCCCGACACTCAATGGCATGAAGTTGTAATTGTTAGTAATCGTG
KJ826432_GEWMA15-16   757 ATTTCAGTTTGGATTTGCCCGACACTCAATGGCATGAAGTTGTAATTGTTAGTAATCGTG
KJ826433_GEWMA61-62   756 ATTTCAGTTTGGATTTGCCCGACACTCAATGGCATGAAGTTGTAATTGTTAGTAATCGTG
KJ826434_GEWMA64      756 ATTTCAGTTTGGATTTGCCCGACACTCAATGGCATGAAGTTGTAATTGTTAGTAATCGTG
consensus             781 *****.******..***.*.****************.******.................
```

```
Bb_B31_IGS              831 TATACCT-TAATATAGAAATTGAATAAATTT-TTGTTTTTCTTATTAATTATAGCTTAAA
Bb_297_IGS              825 TATACCT-TAATATAGAAATTGAATAAATTT-TTGTTTTTCTTATTAATTATAGCTCAAA
Bb_N40_IGS              838 TATACCT-TAATATAGAAATTGAATAAATTT-TTGTTTTTCTTATTAATTATAGCTTAAA
Bb_SGE03-1_IGS          839 TATACCT-TAATATAGAAATTGAATAAATTT-TTGTTTT-----TTTATTATAGCTTAAA
KJ826414_bwtx17         823 TATACCT-TAATATAGAAATTGAATAAATTT-TTGTTTTTCTTATTAATTATAGCTTAAA
KJ826415_IGRFTX4            ------------------------------------------------------------
KJ826416_bwtx32         820 TATACTGTAATAT-AGTGATCAAATAAATCATTGTATCTCATAATCAGTCATCGCTTAAG
KJ826417_bwtx23         817 TATACCT-TAATATAGAAATTGAATAAATTT-TTGTTTTTCTTATTAATTATAGCTTAAA
KJ826418_bwtx31         815 TATACCCTTGATATAGAAAATGAATAAATTT-TTGTTTTTCTTATTAATTATAGCTTAAA
KJ826419_bwtx24         792 TATACCT-TAATATAGAAATTGAATAAATTT-TTGTTTTTCTTATTAATTATAGCTTAAA
KJ826420                815 TATACCT-TAATATAGAAATTGAATAAATTT-TTGTTTTTCTTATTAATTATAGCTTAAA
KJ826421_GEWMA35-36     815 TATACCT-TA-TATAGAAATTGAATAAATTT-TTGTTTTTCTTATAGTTATAGCTTAGAA
KJ826422_GEWMA33        814 TATACCT-TAATATAGAAATTGAATAAATTT-TTGTTTTTCTTATTAATTATAGCTTAAA
KJ826423_CVM5           818 TATACCT-TAATATAGAAATTGAATAAATTT-TTGTTTTTCTTATTAATTATAGCTTAAA
KJ826424_KWMA11         815 TATACCT-TAATATAGAAATTGAATAAATTT-TTGTTTTTCTTATTAATTATAGCTTAAA
KJ826425_LMR2           819 TATACCT-TAATATAGAAATTGAATAAATTT-TTGTTTTTCTTATTAATTATAGCTTAAA
KJ826426_DTX            814 TATACCT-TAATATAGAAATTGAATAAATTT-TTGTTTTTCTTATTAATTATAGCTTAAA
KJ826427_GEWMA38        815 TATACCT-TAATATAGAAATTGAATAAATTT-TTGTTTTTCTTATTAATTATAGCTTAAA
KJ826428_GEWMA12        821 TATACCT-TAATATAGAAATTGAATAAATTT-TTGTTTTTCTTATTAATTATAGCTTAAA
KJ826429_GEWMA4         817 TATACCT-TAATATAGAAATTGAATAAATTT-TTGTTTTTCTTATTAATTATAGCTTAAA
KJ826430.1_GEWMA9       817 TATACCT-TAATATAGAAATTGAATAAATTT-TTGTTTTTCTTATTAATTATAGCTTAAA
KJ826431_LMR1           819 TATACCT-TAATATAGAAATTGAATAAATTT-TTGTTTTTCTTATTAATTATAGCTTAAA
KJ826432_GEWMA15-16     817 TATACCT-TGATATAGAAATTGAATAAATTT-TTGTTTTTCTTATTAATTATAGCTTAGA
KJ826433_GEWMA61-62     816 TATACCT-TAATATAGAAATTGAATAAATTT-TTGTTTTTCTTATTAATTATAGCTTAAA
KJ826434_GEWMA64        816 TATACCT-TAATATAGAAATTGAATAAATTT-TTGTTTTTCTTATTAATTATAGCTTAAA
consensus               841 ............................................................
```

```
Bb_B31_IGS              889 ACAGTATTGTCGTAATTAAAACAATGG-AATACATTGGGACC-AGGATGAGTTGAACA--
Bb_297_IGS              883 ACAGTATTGTCGTAATTAAAACAATGG-AATACATTGGGACC-AGGATGAGTTGAACA--
Bb_N40_IGS              896 ACAGTATTGTCGTAATTAAAACAATGG-AATACATTGGGACC-AGGATGAGTTGAACA--
Bb_SGE03-1_IGS          892 ACAGTATTGTCGTAATTAAAACAATGG-AATACATTGGGACC-AGGATGAGTTGAACA--
KJ826414_bwtx17         881 ACAGTATTGTCGTAATTAAAACAATGG-AATACATTGGGACC-AGGATGAGTTGAACATC
KJ826415_IGRFTX4            ------------------------------------------------------------
KJ826416_bwtx32         879 ACCGTATTGACGTCAATTAAAACACGGGAATACATTGGGACA-AGGATGAGTTGGACATC
KJ826417_bwtx23         875 ACAGTATTGTCGTAATTAAAACAATGG-AATACATTGGGACC-AGGATGAGTTGAACATC
KJ826418_bwtx31         874 ACAGTATTGTCGTACTTAAAACAATGGGATTACTTGGGGACC-AGGATGAGTTGAACATC
KJ826419_bwtx24         850 ACAGTATTGTCGTAATTAAAACAATGG-AATACATTGGGACC-AGGATGAGTTGAACATC
KJ826420                873 ACAGTATTGTCGTAATTAAAACAATGG-AATACATTGGGACC--GGATGAGTTGAACATC
KJ826421_GEWMA35-36     872 --AGTATTGTCGTAT---TATACATGG-AATACATGGGGACCGAGGA---GTTGAACATC
KJ826422_GEWMA33        872 ACAGTATTGTCGTAATTAAAACAATGG-AATACATTGGGACC-AGGATGAGTTGACATCC
KJ826423_CVM5           876 ACAGTATTGTCGTAATTAAAACAATGG-AATACATTGGGACC-AGGATGAGTTGAACATC
KJ826424_KWMA11         873 ACAGTATTGTCGTAATTAAAACAATGG-AATACATTGGGACC-AGGATGAGTTGAACATC
KJ826425_LMR2           877 ACAGTATTGTCGTAATTAAAACAATGG-AATACATTGGGACCAAGGATGAGTTGAACATC
KJ826426_DTX            872 ACAGTATTGTCGTAATTAAAACAATGG-AATACATTGGGACC-AGGATGAGTTGACATCC
KJ826427_GEWMA38        873 ACAGTATTGTCGTAATTAAAACAATGG-AATACATTGGGACC-AGGATGAGTTGAACATC
KJ826428_GEWMA12        879 ACAGTATTGTCGTATTTAAA-CAATGG-AATACATTGGGACC-AGGATGAGTTGAACATC
KJ826429_GEWMA4         875 ACAGTATTGTCGTAATTAAA-CAATGG-AATACATTGGGACC-AGGATGAGTTGAACATC
KJ826430.1_GEWMA9       875 ACAGTATTGTCGTAATTAAAACAATGG-AATACATTGGGACC-AGGATGAGTTGACATCC
KJ826431_LMR1           877 ACAGTATTGTCGTAATTAAAACAATGG-AATACATTGGGACCAAGGATGAGTTGAACATC
KJ826432_GEWMA15-16     875 ACAGTATTGTCGTAATTAAAACAATGG-AATACATTGGGACC-AGGATGAGTTGAACATC
KJ826433_GEWMA61-62     874 ACAGTATTGTCGTAATTAAAACAATGG-AATACATTGGGACC-AGGATGAGTTGACATCC
KJ826434_GEWMA64        874 ACAGTATTGTCGTAATTAAAACAATGG-AATAGATTGGGACC-AGGATGAGTTGAACATC
consensus               901 ............................................................
```

```
Bb_B31_IGS                   ---------------------------
Bb_297_IGS                   ---------------------------
Bb_N40_IGS                   ---------------------------
Bb_SGE03-1_IGS               ---------------------------
KJ826414_bwtx17          939 CGA-CTAGTTGTTATTACCCACAAAA-
KJ826415_IGRFTX4             ---------------------------
KJ826416_bwtx32          938 CCGACCTCTGGGTAAACAAAACAGGCA
KJ826417_bwtx23          933 CGA-CCTCAGTTTTTATCAGAACA---
KJ826418_bwtx31          933 CCA-CCCCAGTTTAATCAAACCGTCAT
KJ826419_bwtx24          908 CG-ACTAG-------------------
KJ826420                 930 CG-ACTAGTTTTTTTTAAACA------
KJ826421_GEWMA35-36      923 CG-ACTCCCGTTCCCACACTCCA----
KJ826422_GEWMA33         930 GAC-TAG--------------------
KJ826423_CVM5            934 CG-ACTCTGTGTATTTCCCCCCCA---
KJ826424_KWMA11          931 CGA-CTAG-------------------
KJ826425_LMR2            936 CG-ACTAGTG-----------------
KJ826426_DTX             930 GCC-G----------------------
KJ826427_GEWMA38         931 CGA-CTAG-------------------
KJ826428_GEWMA12         936 CG-------------------------
KJ826429_GEWMA4          932 CGA-CTCAG------------------
KJ826430.1_GEWMA9        933 GCC-AG---------------------
KJ826431_LMR1            936 CG-ACTAGTG-----------------
KJ826432_GEWMA15-16      933 CGA-CTCGTG-----------------
KJ826433_GEWMA61-62      932 G--------------------------
KJ826434_GEWMA64         932 CGA-CTAG-------------------
consensus                962    ..    ...................
```
